# Epigenetic Regulators Modulate Muscle Damage in Duchenne Muscular Dystrophy Model

**DOI:** 10.1371/currents.md.f1e2379fa632f8135577333dd92ca83b

**Published:** 2017-12-21

**Authors:** Fernanda Bajanca, Laurence Vandel

**Affiliations:** Centre de Biologie du Développement (CBD), Centre de Biologie Intégrative (CBI), Université de Toulouse, CNRS, UPS, France; Centre de Biologie du Développement (CBD), Centre de Biologie Intégrative (CBI), Université de Toulouse, CNRS, UPS, France; Université Clermont Auvergne, CNRS, Inserm, GReD, F-63000 Clermont-Ferrand, France (present address)

## Abstract

Histone acetyl transferases (HATs) and histone deacetylases (HDAC) control transcription during myogenesis. HDACs promote chromatin condensation, inhibiting gene transcription in muscle progenitor cells until myoblast differentiation is triggered and HDACs are released. HATs, namely CBP/p300, activate myogenic regulatory and elongation factors promoting myogenesis. HDAC inhibitors are known to improve regeneration in dystrophic muscles through follistatin upregulation. However, the potential of directly modulating HATs remains unexplored. We tested this possibility in a well-known zebrafish model of Duchenne muscular dystrophy. Interestingly, CBP/p300 transcripts were found downregulated in the absence of Dystrophin. While investigating CBP rescuing potential we observed that dystrophin-null embryos overexpressing CBP actually never show significant muscle damage, even before a first regeneration cycle could occur. We found that the pan-HDAC inhibitor trichostatin A (TSA) also prevents early muscle damage, however the single HAT CBP is as efficient even in low doses. The HAT domain of CBP is required for its full rescuing ability. Importantly, both CBP and TSA prevent early muscle damage without restoring endogenous CBP/p300 neither increasing follistatin transcripts. This suggests a new mechanism of action of epigenetic regulators protecting dystrophin-null muscle fibres from detaching, independent from the known improvement of regeneration upon damage of HDACs inhibitors. This study builds supporting evidence that epigenetic modulators may play a role in determining the severity of muscle dystrophy, controlling the ability to resist muscle damage. Determining the mode of action leading to muscle protection can potentially lead to new treatment options for muscular dystrophies in the future.

## INTRODUCTION

There is no cure to date for muscular dystrophies caused by absent or malfunctioning Dystrophin, be it lethal Duchenne or milder Becker type. Current therapies aim at alleviating the symptoms and delaying the progression of a disease that can be life threatening. One promising pharmacological treatment is to inhibit histone deacetylases (HDACs) 1. HDACs can regulate gene transcription in muscle progenitor cells, by controlling the activity of myogenic regulatory factors and MEF2 family proteins 2^,^^3^. HDACs promote chromatin condensation, inhibiting gene transcription until myoblast differentiation is triggered and HDACs are released. Similarly, blocking HDACs leads to hyper acetylated chromatin, inhibiting condensation and therefore facilitating transcription. HDAC inhibitors ameliorate the dystrophic phenotype by promoting myogenesis and improving regeneration in dystrophic muscles 1^,^^4^^,^^5^. Several studies show that follistatin upregulation by HDAC inhibitors is responsible for boosting regeneration in dystrophic muscles 6^,^^7^ . While studies have been focusing on blocking HDACs to promote hyper acetylated chromatin and transcription, the potential of directly modulating histone acetyl transferases (HATs) on a muscle dystrophy context remains unexplored. CBP (CREB Binding Protein) is a nuclear transcriptional co-activator with HAT activity, belonging to the p300/CBP family 8. It is ubiquitously expressed and acetylates both histone and non-histone proteins to regulate transcription. CBP was shown to functionally activate MyoD by acetylation and to directly interact with MEF2C 5. CBP expression in zebrafish muscle was reported recently 9. We used the zebrafish model, extensively characterised and widely used for studying muscular dystrophy 10, to explore the effects of overexpressing CBP in a Dystrophin-null background. We have found that overexpressing this single HAT rescues the dystrophic phenotype as efficiently as blocking HDACs. Moreover, we observed that both treatments inhibit the early appearance of dystrophic fibres, prior to any effect on regeneration could take place. We report a follistatin-independent mechanism protecting against fibre damage.

## RESULTS

**Figure 1. Epigenetic regulators in zebrafish dystrophic embryos.**
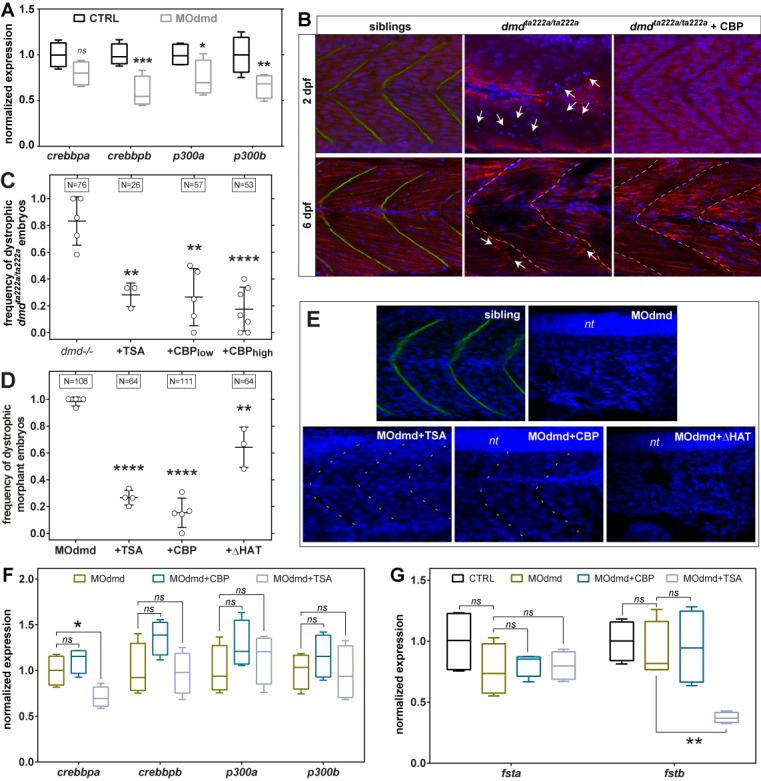
**A)** Quantitative PCR analysis of p300/CBP transcript expression at 2 days post fertilisation (dpf) in control and morpholino-depleted embryos (MOdmd).** (B)** Confocal sections of *Tg(actc1b:mCherry)^pc4^* embryos immunostained for Dystrophin (green). Well organised pattern of muscle fibres (mCherry, red) and nuclei (DAPI, blue) in siblings, while heavily disrupted in *dmd^ta222a/ta222a^* embryos (arrows), showing collapsed and wavy fibres which ends misalign with somatic borders (dashed lines). Overexpressing CBP (*dmd^ta222a/ta222a^* + CBP; injection of 10^7^ RNA molecules of plasmid per embryo at one cell stage) has a clear positive effect on dystrophic embryos both at 2 and 6 dpf.** (C, D)** Frequency of dystrophic phenotype (see Materials and Methods) on 2 dpf Dystrophin-*null* embryos, either *dmd^ta222a/ta222a^*mutants (C) or depleted with MOdmd (D). Overexpressing CBP (low = 10^7^, high = 10^8^ molecules of plasmid RNA injected per embryo) rescues the dystrophic phenotype of both *dmd^ta222a/ta222a^*(C) and morphant (D; 10^7^ molecules) embryos as efficiently as blocking HDACs (TSA), even at lower concentrations (C,D). A mutated CBP form lacking the HAT domain (ΔHAT; 10^7^ molecules) significantly decreases the rescue efficiency (C). Individual values for independent experiments are plotted, bars represent the average ± SD and boxes above plots indicate total number of embryos analysed. **(E)** Representative confocal sections of 2 dpf embryos immunostained for Dystrophin (green) to confirm Dystrophin presence in siblings and depletion in MOdmd embryos, counterstained with DAPI (blue). Both TSA (200 nM) and CBP (10^7^ molecules) are able to rescue the muscle phenotype of MOdmd embryos, while ΔHAT injected embryos most often show muscle damage (10^7^ molecules). nt = neural tube. **(F,G)** Quantitative PCR analysis of *crebbp*, *p300* and *follistatin* endogenous transcripts expression at 2 dpf. See text for details on ANOVA (A,F,G) or *T* tests (B,C), performed with cut-offs: (*) < 0.05, (**) < 0.005, (***) < 0.001, (****) < 0.0001. ns = non significant.

To test whether Dystrophin absence affects HAT's expression we performed quantitative real-time PCR (qPCR) for *p300* and/or *CBP* (*crebbp*) transcripts (Figure 1A). Two-way ANOVA shows highly significant differences [F(1,24)=31.5, p<0.0001] between HAT genes expression in controls and embryos injected with well described Dystrophin morpholino cocktail (also see Materials and methods, S1 Fig A). Multiple comparisons indicate strong downregulation of *crebbpb* and *p300b* and milder but significant decrease of *p300a* expression (*p* values: *crebbpb* = 0.0010, *p300a* = 0.0247, *p300b* = 0.0045). Therefore, the absence of Dystrophin significantly affects HAT expression.

Taking these results, we set to determine whether the dystrophic phenotype could be rescued by overexpressing RFP-tagged murine CBP in *dystrophin*-null embryos (*dmd^ta222a/ta222a^*). Nuclear and dose dependent expression of the transgene was confirmed (S1 Fig B,C). To test the hypothesis that CBP overexpression improves regeneration, embryos were analysed at 2 dpf, as fibre tips start detaching from somitic borders 10, and 6 dpf, when regeneration upon damage is underway 11^,^^12^. While *dystrophin*-null embryos show characteristic dystrophic muscles from early stages, overexpressing CBP clearly reduced damage not only at later stages but, unexpectedly, also at 2 dpf (Figure 1B). Fast swimming was also restored, a characteristic failure of dystrophic embryos. Absence of damage as early as 2 dpf cannot be explained by stimulating regeneration, which takes several days 11^,^^12^, suggesting that CBP overexpression may act at an earlier phase preventing muscle degeneration in the first place. We focused on investigating this observation further and testing whether only CBP overexpression or also HDAC inhibition could have a protective effect. Different concentrations of CBP were tested and rescue at 2 dpf compared with TSA treatment, previously used in zebrafish and considered the most efficient pan-HDAC inhibitor 1^,^^13^ (Figure 1C-E; S1 Fig D). On average, 83% of *dystrophin*-null (*dmd^ta222a/ta222a^*) embryos showed clear dystrophic muscles at 2 dpf (Figure 1C, *dmd^-/-^*). The prevalence of a dystrophic signature was drastically reduced to 28% upon treatment with TSA and 27% when a low dose of CBP (+CBP_low_) was used. A 10-fold increase of the CBP dose (+CBP_high_) improves the rescue significance (p<0.0001). Therefore, CBP overexpression is at least as efficient as HDACs inhibition in protecting *dystrophin*-null muscles from damage. Similar results were obtained in the Dystrophin morphant background for both HDACs inhibitor and CBP (Figure 1D,E). Immunostaining for RFP-CBP shows that rescue is obtained in morphants even when detectable levels are seen in few muscle nuclei (S1 Fig D). Overexpression of a CBP form lacking active HAT domain leads to mild rescue indicating that the acetylase activity of CBP is essential for full rescue (+ΔHAT; Figure 1D,E).

Since CBP/p300 expression is generally downregulated in Dystrophin-depleted embryos (Figure 1A), we tested whether CBP overexpression or TSA act though increasing endogenous *crebbp/p300* levels. Dystrophin-depleted embryos were co-injected with CBP (10^7^ molecules) or treated with TSA and qPCR was performed at 2 dpf (Figure 1F; S1 Fig E). One-way ANOVA revealed statistically significant differences between treatment groups for *crebbpa* expression [F (2, 9) = 8.915, p = 0.0073**], Fisher's LSD post-hoc test finding the decrease upon TSA treatment statistically significant (p = 0.0164*). Independent one-way ANOVAs revealed absence of statistically significant differences in the expression of *crebbpb* [F (2, 9) = 3.340, p = 0.0823], *p300a* [F (2, 9) = 1.099, p = 0.3741] and *p300b* [F (2, 9) = 0.6550, p = 0.5425] transcripts. These results show that neither CBP nor TSA significantly increase any of the transcripts, *crebbpa* being even decreased upon TSA treatment (Figure 1F). Therefore, rescue is not due to restoring endogenous CBP/p300 transcripts levels.

While the mechanism of action of HDAC inhibition leading to improved regeneration is not fully understood, follistatin upregulation is a key event 6^,^^7^. We questioned whether the follistatin pathway could also be involved in the early protection against muscle damage observed in TSA treatment and CBP overexpression. Quantitative analysis of the two zebrafish follistatin transcripts, *fsta* and *fstb*, expression was performed at 2 dpf (Figure 1G). Dystrophin depletion does not significantly affect *fsta* or *fstb* levels. There were no statistically significant differences in the expression of *fsta* between groups as determined by one-way ANOVA [F (3, 12) = 1.288, p = 0.3232]. The expression of *fstb* is statistically significantly different between groups [F (3, 12) = 7.680, p = 0.0040**], Fisher's LSD post-hoc test finds the decrease upon TSA treatment statistically significant (p = 0.0013**). Summarising, TSA treatment significantly downregulates the expression of *fstb* while not affecting *fsta* and both transcripts remain unaltered by CBP overexpression. Therefore, the protection against muscle damage granted by CBP overexpression or TSA treatment does not rely on stimulating follistatin transcription but likely on a follistatin-independent pathway.

## FINAL REMARKS

This study shows for the first time that either CBP overexpression or pan-HDAC inhibition are able to prevent, or at least delay, early stages of muscle damage in zebrafish dystrophic muscles. This observation adds up to the recognised positive effect of HDAC inhibitors on muscle regeneration upon damage 1. Other authors observed that treating mouse dystrophic muscles (mdx) with deacetylase inhibitors was able to confer resistance to contraction-coupled degeneration 14. However, this was suggested to be mediated by follistatin through satellite cell number increase. We show that damage protection occurs independently from regeneration. Follistatin upregulation is not involved in protecting early zebrafish muscle from damage, supporting the idea that the two rescuing mechanisms triggered by HDAC inhibition are independent. That the effect of TSA on *follistatin* expression differs according to the cellular context is not surprising. What we report here is a muscle-cell autonomous effect of TSA preventing degeneration, and in this context we observed a downregulation of *follistatin*. Other authors focused on a later effect on regeneration of already damaged muscles, mediated by satellite cells stimulation 1^,^^5^^,^^6^. In this context, follistatin was found to be upregulated. However, it was shown that TSA promotes follistatin upregulation in the fibroadipogenic progenitors rather than in the muscle satellite cells, and the stimulating effect is paracrine 7^,^^15^. Interestingly, overexpression of a single HAT, CBP, achieves a similar level of protection as pan-HDAC inhibition. The strong downregulation of *fstb* by TSA but not CBP should be investigated in future work to determine whether these agents may act through different mechanisms of action. Our results prompt for investigating similar mechanisms in other animal models and identifying new therapeutic targets, possibly with more restricted and controlled effects than HDAC inhibitors. This study strengthen the idea that epigenetics plays a role in the progression of symptoms in some patients with Dystrophin mutations. This could explain the variation in severity in Becker muscular dystrophy patients with identical mutation and the variable progression of DMD. Doubt persists on the mechanism leading to protection. To further understand the mechanisms of action triggered by either HDAC inhibition or CBP a high throughput analysis needs to be employed in future studies.

## MATERIALS AND METHODS

**Animals**

Fish used were wild-type Danio rerio, *dmd^ta222a/+^* and *dmd^ta222a/+^* crossed to *Tg(actc1b:mCherry)^pc4^* to facilitate identifying dystrophic muscles. All animals were handled in a facility certified by the French Ministry of Agriculture (approval ID A-31-555-01) and in accordance with the guidelines from the European directive on the protection of animals used for scientific purposes (2010/63/UE), French Decret 2013–118. Staging and rearing was performed as described 16.

**Plasmids, morpholinos and HDACs inhibition**

pCS2+mRFP-CBP and pCS2+mRFP-ΔHAT plasmids were created by digesting by BamH1 full length CBP of mouse origin, or CBP deleted for amino acids 1430 to 1570 from pCMV-HA-CBP and pCMV-HA-CBPΔHAT (provided by A. Harel-Bellan) and inserting them into pCS2+mRFP-N1 vector (Addgene) digested by BamH1. To produce capped RNA to inject, the vectors were linearized with NotI and the mMESSAGE mMACHINE SP6 kit (Ambion) was used. Injections were performed at one-cell stage with the indicated amounts of *RFP-CBP* and *RFP-CBPΔHAT* RNA. RNA copy number was calculated using the NEBioCalculator v1.7.1 (BioLabs). Mouse CBP shares 85% protein identity to both zebrafish CBP-A (the product of *crebbpb*) and zebrafish CBP-B (the product of *crebbpa*), according to the standard NCBI BLAST tools. Zebrafish protein CREBBPA (also called CREBBPb or CBP-B) corresponds to protein ID ENSDARP00000081684, while CREBBPB protein ID (also called CREBBPa or CBP-A) corresponds to ENSDARP00000086306.

Morpholinos were from Gene-tools and were used as a cocktail as described previously (*dmd*-MO1 at 4 ng per embryo and *dmd*-MO6 at 7.5ng/embryo 13.

Embryos were exposed to Trichostatin A (TSA, Sigma) for 24 hours, from 24 to 48 hpf. TSA was added to fish water for a final concentration of 200 nM as described previously 13^,^^17^.

**Frequency of dystrophic embryos and rescue analysis**

Phenolthiourea (0.003%) was added at 24 hpf to inhibit pigmentation and facilitate muscle analysis. To determine the dystrophic phenotype frequency each embryo was dechorionated individually and observed free moving, then anaesthetized with tricaine (0.2 mg/ml) to facilitate close observation of muscles. The embryos were split into two groups, “normal” or “dystrophic” phenotype. Dystrophic muscles were characterised by abnormal birefringence, supported by observing disruption of the actin reporter pattern when *Tg(actc1b:mCherry)^pc4^* fish were used, and impaired movement 10^,^^18^. After sorting by phenotype, immunohistochemistry (details below) was performed to identify embryos negative for Dystrophin expression (*i.e.*, *dmd^ta222a/ta222a^*or *dmd*-morphants) within both the “normal” and “dystrophic” phenotypical groups. The plotted “frequency of dystrophic embryos” refers to the relative amount of Dystrophin-negative embryos that effectively show a dystrophic phenotype. Rescued embryos are those that despite being Dystrophin-negative do not show a dystrophic phenotype upon a specific treatment. Note that the rescue analysis is blind in the sense that the phenotype analysis is done prior to any possibility of knowing the genotype of the embryo, or the state of Dystrophin expression.

**Immunohistochemistry**

Standard protocols were used. Embryos were fixed in cold methanol for dystrophin staining, or otherwise in paraformaldehyde 4%. Primary antibodies were mouse anti-Dystrophin (MANDRA1 (7A10), Developmental Studies Hybridoma Bank) and rat anti-RFP (Chromotek). Secondary antibodies were goat anti-mouse Alexa-488 and goat anti-rat Alexa-546 (Molecular Probes). DAPI was used to counterstain nuclei.

**Microscopy, software and statistics**

An inverted Zeiss 710 LSM with a 20x/1.0 W Plan-Apochromat and a 40x/1.3 Plan-Apochromat objective were used for Z-stack acquisition. Acquisition and maximum intensity projections were made with ZEN 2009/2010 (Zeiss). Images were uniformly contrasted with Adobe Photoshop CS6. Illustrations were made in Adobe Illustrator CS6. GraphPad Prism 6 was used for graph plotting and statistical analyses.

**Real time quantitative PCR**

Four samples were analysed for each condition, each sample consisting of 15 embryos at 2 days post fertilisation (dpf) for a total of 60 embryos per condition. Total RNAs were extracted with the EZNA total RNA kit I (Omega Bio-tek), and reverse-transcribed with the qScript cDNA synthesis kit (Quanta biosciences) according to the supplier’s instructions. A C1000™ Thermal Cycler with CFX96™ Real time System (BioRad) was used to perform the qPCR and analyses were performed on BioRad CFX Manager 2.0, according to the manufacturer’s instructions. SsoFast™ EvaGreen® Supermix (Bio-Rad) was used according to the manufacturer’s instructions. The following primer sequences were used: *EF1α* Fwd 5’- GAT GCA CCA CGA GTC TCT GA -3’; *EF1α* Rev 5’- TGA TGA CCT GAG CGT TGA AG -3’; m-*crebbp* Fwd 5'- TGC CAA GTT GCC CAT TGT G -3'; m-*crebbp* Rev 5'- TTG TTG GTT TCG CTT GTC ACT -3' ; z-*crebbpa* Fwd 5’- CGA AAA GTG GAA GGG GAC AT -3’; z-*crebbpa* Rev 5’- TTC TCT TCC AGC TCT TTC TGG -3’; z-*crebbpb* Fwd 5’- CAG GTT CCT CAA GGG ATG G -3’; z-*crebbpb* Rev 5'- CCA TCA TGG CTT GAG CTT G -3’; *p300a* Fwd 5'- CAC CTT CCT CAA CAC CAC AGT -3’; *p300a* Rev 5'- GCA TAG CAT TCT GGT CTG CTC -3'; *p300b* Fwd 5'- ATA TGG CCG TCA GGG TTT ATC -3'; *p300b* Rev 5'- CTC GTG TCT CCA GAA AGT TGC -3'; *fsta* Fwd 5’- GAT GCA AAA TGA ACA GGA GGA -3’; *fsta* Rev 5’- GAC TTC AAA AGG GCA CAT TCA -3’; *fstb* Fwd 5’- GCA TGG ACT GAG GAG GAT GTA -3’; *fstb* Rev 5’- CAC CTC TTT CCA GAA CCA CAA -3’. All experiments included a standard curve for each primer pair and control for genomic DNA contamination. Data were normalized to *EF1α* and expressed relative to the respective control that is set as 1 in boxplot with whiskers representing the median, 0.25 percentiles, maximum and minimum values.

## Funding

This work was supported by grants from the Fondation de l’Association pour la Recherche contre le Cancer (Fondation ARC ; https://www.fondation-arc.org/arc-foundation; SFI20121205590); Association Française contre les Myopathies (AFM ; http://www.afm-telethon.com; 20102); joint call Alliance pour la Vie et pour la Santé (AVIESAN ; https://aviesan.fr) et Plan Cancer (www.plan-cancer.gouv.fr) (P036407) and Institut National du Cancer (INCa ; http://www.e-cancer.fr; P030972) to L.V and a fellowship from the Fondation pour la Recherche Médicale (FRM ; https://www.frm.org ; SPF20130526627) to F.B.

## Competing Interests

The authors have declared that no competing interests exist.

## Data Availability

The values behind statistics and graphs presented here are deposited at FigShare, DOI: 10.6084/m9.figshare.5570038.

## Corresponding Authors

Fernanda Bajanca, fbajanca@gmail.com

Laurence Vandel, laurence.vandel@uca.fr

## Supplementary Information

**Figure:**
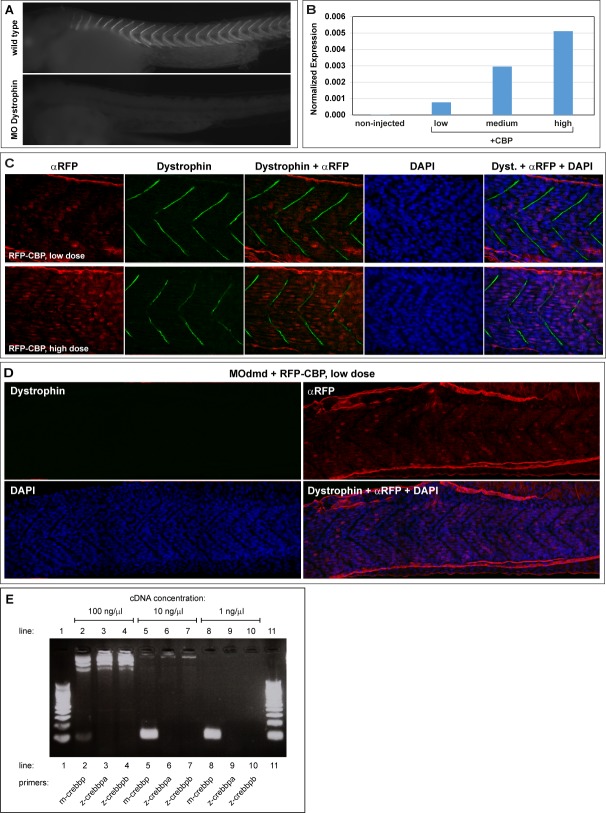
**S1 Fig. (A)** Wild-type and MOdmd injected zebrafish embryos at 2 dpf, showing drastic reduction of Dystrophin on embryos injected with morpholino cocktail compared with wild type expression. No secondary developmental defects were observed. **(B)** Control quantitative PCR with specific primers for *crebbp* of mouse origin performed on 2 dpf control embryos (non-injected) and embryos injected at one-cell stage with 10^7^ (low), 5x10^7^ (medium) or 10^8^ (high) molecules of mRFP-CBP. Note that: i) endogenous zebrafish *crebbp* transcripts are not detected in control non-injected embryos; ii) the number of transcripts detected at 2 dpf increases linearly with the increasing number of transcripts injected at one cell stage. **(C)** Representative confocal sections of 2 dpf wild type embryos injected at one-cell stage with 10-fold doses of RFP-tagged CBP: 10^7^ (low) and 10^8^ (high) RNA molecules per embryo. Embryos were immunostained for exogenous CBP (αRFP; red), Dystrophin (green), and nuclei counterstained with DAPI (blue). Note the distribution of exogenous CBP, accumulated in the nuclei of a small subset of muscle cells when a lower dose is injected (B, red) compared with more widespread expression when a higher dose is injected (C, red).** (D)** Representative confocal tiled section of a 2 dpf embryo, co-injected at one-cell stage with MOdmd plus 10^7^ RNA molecules (low dose) of RFP-tagged CBP expression plasmid. Panels show immunostaining for Dystrophin (green), exogenous CBP (αRFP; red) and nuclei counterstained with DAPI (blue). Note that despite Dystrophin expression is undetectable the muscles are not damaged, even if only a small subset of total nuclei express exogenous CBP. **(E)** Control for z-*crebbpa* and z-*crebbpb* qPCR primers specificity: neither detects the exogenous CBP of mouse origin. PCR was performed on template pCS2+mRFP-CBP cDNA at concentrations: 100 ng/µl (lines 2-4), 10 ng/µl (lines 5-7) and 1 ng/µl (lines 8-10), and primers: m-*crebbp* (lines 2, 5, 8), z-*crebbpa* (lines 3, 6, 9) and z-*crebbpb* (lines 4, 7, 10). A 100-bp ladder (Biolabs) was used on lines 1 and 11.

## References

[1] Consalvi S, Saccone V, Giordani L, Minetti G, Mozzetta C, Puri PL. Histone deacetylase inhibitors in the treatment of muscular dystrophies: epigenetic drugs for genetic diseases. Mol Med. 2011;17: 457–65. doi:10.2119/molmed.2011.00049 10.2119/molmed.2011.00049 PMC3105131 21308150

[2] Palacios D, Puri PL. The epigenetic network regulating muscle development and regeneration. J Cell Physiol. 2006;207: 1–11. doi:10.1002/jcp.20489 10.1002/jcp.20489 16155926

[3] McKinsey TA, Zhang CL, Olson EN. Activation of the myocyte enhancer factor-2 transcription factor by calcium/calmodulin-dependent protein kinase-stimulated binding of 14-3-3 to histone deacetylase 5. Proc Natl Acad Sci. 2000;97: 14400–14405. doi:10.1073/pnas.260501497 10.1073/pnas.260501497 PMC18930 11114197

[4] Iezzi S, Cossu G, Nervi C, Sartorelli V, Puri PL. Stage-specific modulation of skeletal myogenesis by inhibitors of nuclear deacetylases. Proc Natl Acad Sci U S A. 2002;99: 7757–62. doi:10.1073/pnas.112218599 10.1073/pnas.112218599 PMC124343 12032356

[5] Giordani L, Puri PL. Epigenetic control of skeletal muscle regeneration: Integrating genetic determinants and environmental changes. FEBS J. 2013;280: 4014–4025. doi:10.1111/febs.12383 10.1111/febs.12383 PMC3753079 23745685

[6] Iezzi S, Di Padova M, Serra C, Caretti G, Simone C, Maklan E, et al. Deacetylase inhibitors increase muscle cell size by promoting myoblast recruitment and fusion through induction of follistatin. Dev Cell. 2004;6: 673–84. doi:10.1016/S1534-5807(04)00107-8 10.1016/s1534-5807(04)00107-8 15130492

[7] Mozzetta C, Consalvi S, Saccone V, Tierney M, Diamantini A, Mitchell KJ, et al. Fibroadipogenic progenitors mediate the ability of HDAC inhibitors to promote regeneration in dystrophic muscles of young, but not old Mdx mice. EMBO Mol Med. 2013;5: 626–639. doi:10.1002/emmm.201202096 10.1002/emmm.201202096 PMC3628105 23505062

[8] Bedford DC, Kasper LH, Fukuyama T, Brindle PK. Target gene context influences the transcriptional requirement for the KAT3 family of CBP and p300 histone acetyltransferases. Epigenetics. 2010;5: 9–15. 10.4161/epi.5.1.10449 PMC2829352 20110770

[9] Batut J, Duboé C, Vandel L. Expression patterns of CREB binding protein (CREBBP) and its methylated species during zebrafish development. Int J Dev Biol. 2015;59: 229–34. doi:10.1387/ijdb.140197LV 10.1387/ijdb.140197LV 26260685

[10] Bajanca F, Gonzalez-Perez V, Gillespie SJ, Beley C, Garcia L, Theveneau E, et al. In vivo dynamics of skeletal muscle Dystrophin in zebrafish embryos revealed by improved FRAP analysis. Elife. 2015;4. doi:10.7554/eLife.06541 10.7554/eLife.06541 PMC4601390 26459831

[11] Gurevich DB, Nguyen PD, Siegel AL, Ehrlich O V., Sonntag C, Phan JMN, et al. Asymmetric division of clonal muscle stem cells coordinates muscle regeneration in vivo. Science (80- ). 2016;353: aad9969-aad9969. doi:10.1126/science.aad9969 10.1126/science.aad9969 27198673

[12] Pipalia TG, Koth J, Roy SD, Hammond CL, Kawakami K, Hughes SM. Cellular dynamics of regeneration reveals role of two distinct Pax7 stem cell populations in larval zebrafish muscle repair. Dis Model Mech. 2016;9: 671–684. doi:10.1242/dmm.022251 10.1242/dmm.022251 PMC4920144 27149989

[13] Johnson NM, Farr GH, Maves L. The HDAC Inhibitor TSA Ameliorates a Zebrafish Model of Duchenne Muscular Dystrophy. PLoS Curr. 2013;5. doi:10.1371/currents.md.8273cf41db10e2d15dd3ab827cb4b027 10.1371/currents.md.8273cf41db10e2d15dd3ab827cb4b027 PMC3870918 24459606

[14] Minetti GC, Colussi C, Adami R, Serra C, Mozzetta C, Parente V, et al. Functional and morphological recovery of dystrophic muscles in mice treated with deacetylase inhibitors. Nat Med. 2006;12: 1147–1150. doi:10.1038/nm1479 10.1038/nm1479 16980968

[15] Saccone V, Consalvi S, Giordani L, Mozzetta C, Barozzi I, Sandona M, et al. HDAC-regulated myomiRs control BAF60 variant exchange and direct the functional phenotype of fibro-adipogenic progenitors in dystrophic muscles. Genes Dev. 2014;28: 841–857. doi:10.1101/gad.234468.113 10.1101/gad.234468.113 PMC4003277 24682306

[16] Westerfield M. The Zebrafish Book - a guide for the laboratory use of zebrafish (Danio rerio). 3 edn. University of Oregon Press; 1995.

[17] He Y, Mei H, Yu H, Sun S, Ni W, Li H. Role of histone deacetylase activity in the developing lateral line neuromast of zebrafish larvae. Exp Mol Med. Nature Publishing Group; 2014;46: e94. doi:10.1038/emm.2014.18 10.1038/emm.2014.18 PMC3972790 24810423

[18] Berger J, Sztal T, Currie PD. Quantification of birefringence readily measures the level of muscle damage in zebrafish. Biochem Biophys Res Commun. Elsevier Inc.; 2012;423: 785–8. doi:10.1016/j.bbrc.2012.06.040 10.1016/j.bbrc.2012.06.040 22713473

